# Cincinnati Academy of Dentistry

**Published:** 1896-08

**Authors:** 


### Cincinnati Academy of Dentistry.

At the regular monthly meeting of the Cincinnati Academy of Dentistry, held Monday evening, April 27, 1896, the following officers were elected for the ensuing year, viz.:

President—W. T. McLean, M.D., D.D.S.
Vice-President—A. I. F. Buxbaum, M.D., D.D.S.
Secretary—Wm. Lockman, jr., D.D.S.
Treasurer—J. F. Clayton, D.D.S.

